# ViDa1: The Development and Validation of a New Questionnaire for Measuring Health-Related Quality of Life in Patients with Type 1 Diabetes

**DOI:** 10.3389/fpsyg.2017.00904

**Published:** 2017-05-31

**Authors:** Dácil Alvarado-Martel, M. Angeles Ruiz Fernández, Maribel Cuadrado Vigaray, Armando Carrillo, Mauro Boronat, Ana Expósito Montesdeoca, Lía Nattero Chávez, Maite Pozuelo Sánchez, Pino López Quevedo, Ana D. Santana Suárez, Natalia Hillman, David Subias, Pilar Martin Vaquero, Lourdes Sáez de Ibarra, Didac Mauricio, Pedro de Pablos-Velasco, Francisco J. Nóvoa, Ana M. Wägner

**Affiliations:** ^1^Department of Endocrinology and Nutrition, Complejo Hospitalario Univesitario Insular Materno-Infantil de Gran CanariaLas Palmas de Gran Canaria, Spain; ^2^Instituto Universitario de Investigaciones Biomédicas y Sanitarias, Universidad de Las Palmas de Gran CanariaLas Palmas de Gran Canaria, Spain; ^3^Universidad Nacional de Educación a DistanciaMadrid, Spain; ^4^Department of Endocrinology and Nutrition, CIBER of Diabetes and Associated Metabolic Diseases, Hospital Universitario Germans Trias i PujolBadalona, Spain; ^5^Department of Endocrinology and Nutrition, Hospital Universitario Ramón y CajalMadrid, Spain; ^6^Department of Endocrinology and Nutrition, Hospital Universitario de Gran Canaria Doctor NegrínLas Palmas de Gran Canaria, Spain; ^7^Unit of Diabetes, Hospital Universitario La PazMadrid, Spain; ^8^Department of Endocrinology and Nutrition, Hospital Universitario Parc TaulíSabadell, Spain; ^9^Unit of Diabetes and Metabolism, D-Médical ClinicMadrid, Spain

**Keywords:** health-related quality of life, type 1 diabetes, questionnaire, validation, patients, patient-reported outcome, patient centered research

## Abstract

This study describes the development of a new questionnaire to measure health-related quality of life (HRQoL) in patients with type 1 diabetes (the ViDa1 questionnaire) and provides information on its psychometric properties. For its development, open interviews with patients took place and topics relevant to patients' HRQoL were identified and items were generated. Qualitative analysis of items, expert review, and refinement of the questionnaire followed. A pilot study (*N* = 150) was conducted to explore the underlying structure of the 40-item ViDa1 questionnaire. A Principal Component Analysis (PCA) was performed and six of the items that did not load on any of the factors were eliminated. The results supported a four-dimensional structure for ViDa1, the dimensions being Interference of diabetes in everyday life, Self-care, Well-being, and Worry about the disease. Subsequently, the PCA was repeated in a larger sample (*N* = 578) with the reduced 34-item version of the questionnaire, and a Confirmatory Factor Analysis (CFA) was performed (*N* = 428). Overall fit indices obtained presented adequate values which supported the four-factor model initially proposed [($\chi_{\left(df=554\right)}^{2}=$ 2601.93) (*p* < 0.001); Root Mean Square Error of Approximation = 0.060 (CI = 0.056 −0.064)]. As regards reliability, the four dimensions of the ViDa1 demonstrated good internal consistency, with Cronbach's alphas ranging between 0.71 and 0.86. Evidence of convergent-discriminant validity in the form of high correlations with another specific HRQoL questionnaire for diabetes and low correlations with other constructs such as self-efficacy, anxiety, and depression were presented. The ViDa1 also discriminated between different aspects of clinical interest such as type of insulin treatment, presence of chronic complications, and glycemic control, temporal stability, and sensitivity to change after an intervention. In conclusion, the ViDa1 questionnaire presents adequate psychometric properties and may represent a good alternative for the evaluation of HRQoL in type 1 diabetes.

## Introduction

Type 1 diabetes is a chronic disease caused by autoimmune destruction of the beta cells in the pancreas, leading to an absolute insulin deficit. People with type 1 diabetes need to inject insulin in order to survive (Atkinson et al., 2014).

The discovery of insulin in 1921 represented a decisive breakthrough in the prognosis of type 1 diabetes, which had previously been a fatal disease. The publication of the results of the Diabetes Control and Complications Trial (DCCT) in the early 1990s was another milestone in the management of type 1 diabetes; intensive treatment with multiple (3–6) injections or a continuous subcutaneous insulin infusion pump in order to reach near normal concentrations of blood glucose was shown to reduce the development of chronic complications and to improve disease control (DCCT Research Group, 1993). Thereafter, intensive insulin therapy became the standard of care in type 1 diabetes.

In addition to the administration of insulin, intensive treatment also requires the frequent measurement of glucose in capillary blood and adjustment of the insulin dose depending on glucose levels, intake, and planned physical activity (DCCT Research Group, 1993). The management of type 1 diabetes is complex and requires important lifestyle changes. Patients have to take responsibility for a large part of their care and make real-time decisions about their treatment schedules and about the doses of insulin they administer several times a day. The complexity and intensity of self-care in diabetes has been reported to interfere with the patient's health-related quality of life (HRQoL; Wolpert and Anderson, 2001; Escudero-Carretero et al., 2007).

HRQoL has been widely studied in people with type 1 diabetes, generally in relation to other aspects such as the presence of chronic complications (DCCT Research Group, 1996; Hahl et al., 2002; Jacobson et al., 2013), glycemic control (Hoey et al., 2001; Tan et al., 2005; Cooke et al., 2015; Stahl-Pehe et al., 2017), disease duration (Sparring et al., 2013) and the impact of structured education programs (Speight et al., 2010; Byrne et al., 2012). The question of how to assess the burden of treatment and its effect on HRQoL in patients with type 1 diabetes has received less attention to date.

Determining the impact of disease on patients' lives is vitally important in clinical practice because it helps clinicians to detect needs, identify barriers to self-care and make clinical decisions, and improve communication with patients (Testa and Simonson, 1996; Huang et al., 2008). Disease-specific instruments for measuring HRQoL have been shown to be more useful than general ones, because they are more sensitive to the changes in the particular disease and provide more detailed information (Testa and Simonson, 1996). To our knowledge, two specific instruments for measuring HRQoL in diabetes have been adapted and validated in Spain: the Audit of Diabetes-Dependent Quality of Life (ADDQoL) and the Diabetes Quality of Life Measure (DQoL).

The DQoL (Jacobson et al., 1988) was the first research instrument to measure HRQoL in diabetes. The DQoL was designed as part of the DCCT study to assess the impact of intensive treatment and complications. A Spanish version, named the EsDQoL, was adapted and validated in the early 2000s (Millán et al., 2002). The EsDQoL has 43 items grouped into four dimensions: satisfaction, impact, social/vocational worry, and diabetes-related worry. Adequate content validity and internal consistency have been reported in studies of patients with type 2 diabetes (Correr et al., 2008; Pakpour et al., 2012) and with type 1 diabetes (Millán et al., 2002).

However, some of its items have low reliability coefficients and many of the items on the social/vocational worry subscale are not applicable to all people (Gibbons and Fitzpatrick, 2009). The DQoL is a long questionnaire and some questions seem conceptually difficult, for example: “*How often do you find yourself explaining what it means to have diabetes?*” or “…*that your body looks different because you have diabetes?*.” Others seem inappropriate: “…*How often do you feel that you have to go the bathroom more than others?*.” And some of the items use obsolete terms or sound outdated: for example, “*insulin reaction*,” or “*typewriter*” (Gibbons and Fitzpatrick, 2009).

As regards its discriminant validity, the DQoL has demonstrated sensitivity for identifying patients with chronic complications (DCCT Research Group, 1996), but not for intensifying insulin treatment (DCCT Research Group, 1996) or when applied to patients receiving continuous subcutaneous insulin infusion (Tsui et al., 2001).

The ADDQol (Bradley et al., 1999) is another widely used instrument. A version in Argentinian Spanish has been adapted and validated (Perrotta and Irazola, 2002) and has been used in several studies of Spanish patients with type 2 diabetes (Botija Yagüe et al., 2007; Alcubierre et al., 2014; DePablos-Velasco et al., 2014). The Spanish version has 19 items that refer to the following areas: leisure activities, work life, travel, vacations, physical health, family life, friends/social life, sex life, physical appearance, self-confidence, motivation, society/other people's reactions, the future, finances, living arrangements, dependence on others, and freedom to eat and drink. Previous analyses of the ADDQoL support a one-dimensional structure for the questionnaire and an adequate internal consistency, with Cronbach's alphas ranging from 0.81 to 0.95. (Bradley et al., 1999; da Costa et al., 2006; Kong et al., 2011; Fung et al., 2016). However, in some of its linguistic adaptations a strict, single-factor structure was not generated (Ostini et al., 2012; Hirose et al., 2016).

The ADDQol has a complex structure, since it measures separately the importance that patients attach to each area and the impact that it has on their lives (Speight et al., 2009). In addition, its items formulate hypothetical situations that the patient may or may not be able to imagine (i.e., what life would be like without diabetes; Gibbons and Fitzpatrick, 2009; Speight et al., 2009). This is a complex cognitive task, especially for patients who were diagnosed as children and who have lived with the disease ever since (type 1 diabetes is usually diagnosed before the age of 14; Atkinson et al., 2014). Indeed, the Food and Drug Administration argues against the use of instruments which elicit hypothetical responses and which require patients to imagine a desired condition rather than their actual one (The Food and Drug Administration, 2006).

The ADDQol has shown validity for discriminating between patients with type 2 diabetes receiving insulin or oral drugs and between those with better or worse glycemic control (Sundaram et al., 2007) and has shown sensitivity to change following a therapeutic education program (DAFNE Study Group, 2002). However, it did not prove sensitive to change in patients with type 1 diabetes participating in a virtual platform designed to facilitate self-management (Alvarado-Martel et al., 2015a).

The DQoL and the ADDQoL are used interchangeably in patients with type 1 and type 2 diabetes. However, therapeutic guidelines in type 1 diabetes are much more complex and demanding and require greater commitment from the patient. Neither, questionnaire makes explicit mention of aspects of self-care which have a bearing on the HRQoL of patients with type 1 diabetes, such as carbohydrate counting and self-measurement of glucose, or their concern about hypoglycemia (Wolpert and Anderson, 2001; Escudero-Carretero et al., 2007; Alvarado-Martel et al., 2015b).

Finally, these instruments were created some 20 or 30 years ago and the concerns and needs of patients with type 1 diabetes have evolved since then. Today, glucose meters with calculation tools are available, insulin regimes are more flexible, access to information is easier thanks to the new technologies, and there is a growing emphasis on patient-centered training. All these factors should be taken into account when measuring HRQoL in people with type 1 diabetes, since the experience of the disease today is unlikely to be the same as when the currently available questionnaires were developed.

In our view there is a need for a new, patient-centered instrument that is suitable for use in both clinical practice and research. We developed a new, specific questionnaire to measure HRQoL in patients with type 1 diabetes: the ViDa questionnaire for type 1 diabetes (ViDa1). Here, we describe its structure, reliability, convergent and discriminant validities, temporal stability, and sensitivity to change in a heterogeneous sample of patients with type 1 diabetes.

## Materials and methods

### Creation of the questionnaire

The development and validation of the instrument consisted of four stages: 1: Identification of topics relevant to patients, and generation of the items, 2: Qualitative analysis of items, expert review, and refinement of the questionnaire, 3: Pilot study, and 4: Psychometric study of the final questionnaire.

#### Stage 1: Identification of topics relevant to patients, and generation of the items

The contents of the instrument were based on the information obtained in a previous qualitative study involving open interviews with 67 people with type 1 diabetes (Alvarado-Martel et al., 2015b). These interviews were designed to allow patients to describe what HRQoL meant to them, and to record their needs and concerns regarding their disease.

The information obtained from these interviews was examined and the answers were grouped together to create the following 19 themes: 1: self-perception of general HRQoL, 2: social and family relationships, 3: leisure and leisure time, 4: limitations on work, 5: sex life, 6: physical activity, 7: chronic complications, 8: physical/psychological well-being, 9: sleep, 10: satisfaction with treatment, 11: glucose measurement, 12: involvement in and satisfaction with glycemic control, 13: acceptance of disease, 14: motivation, 15: flexibility in diet, 16: satisfaction with the level of knowledge of diabetes, 17: daily stress caused by self-care, 18: fear of/worry about hypoglycemia, and 19: worry about hyperglycemia.

A qualitative analysis of the themes showed that many of them were interrelated and could be grouped *a priori* into a series of broader categories: General quality of life, or Well-being (self-perception of general HRQoL, physical activity, physical/psychological well-being, sleep), Self-care of diabetes (satisfaction with treatment, glucose measurement, involvement and satisfaction with glycemic control, motivation, flexibility in diet, satisfaction with one's knowledge of the disease), Interference of diabetes in the person's daily life and limitations (social and family relations, leisure and free time, limitations on work, sex life, chronic complications, acceptance of illness, daily stress caused by self-care) and Worry about chronic and acute complications of the disease (fear of/worry about hypoglycemia, worry about hyperglycemia and chronic complications). This theme grouping into four defined categories constituted our initial theoretical hypothesis.

Based on these 19 themes, a list of 53 disease-related issues was identified (e.g., *flexibility in my diet, acceptance of my diabetes*) that reflected patients' attitudes and behaviors (Table 1). All issues were evaluated by 10 different patients who indicated on a Likert scale (1 = “none at all” to 10 = “a great deal”) the importance that they attached to them in their daily lives. A descriptive analysis indicated that all were considered important.

**Table 1:** Issues on the Health-Related Quality of Life Questionnaire.

1	Responsibility for my diabetes
2	Motivation for self-care of my diabetes
3	Involvement in the care of my diabetes
4	Autonomy in the management of my diabetes
5	The staff who take care of me should have knowledge of diabetes
6	The time spent on the care of diabetes
7	Level of knowledge of/training in diabetes
8	The adjustment of insulin to the diet
9	Pharmacological treatment
10	Flexibility in the diet
11	Everyday stress due to care duties
12	The feeling of being different
13	Limitations on food
14	Eating out
15	Physical activity/exercise
16	Limitations on work and professional activity due to diabetes
17	Being seen differently or treated differently because of my diabetes
18	Satisfied with glycemic control
19	Leisure time for doing things that I like
20	The fact that others know I have diabetes
21	Injecting insulin
22	Constantly thinking about how I am feeling, about my level of glycemia
23	Carrying out the glucose measurements
24	Fear about having hypoglycemias
25	The possible complications of the illness
26	The distress caused by this illness
27	My sex life
28	My sleep, the time I rest and sleep
29	Feeling good physically
30	My physical appearance
31	Feeling good psychologically
32	Being limited by diabetes and/or its complications
33	Having a good relationship with the health staff who take care of my diabetes
34	Worry about third parties such as partner/children
35	My relationship with my family
36	My relationship with friends/acquaintances/colleagues at work
37	The fact that the people around me are familiar with diabetes
38	Feeling judged by the people around me
39	Being in good health
40	Feeling limited due to my health
41	Being able to carry out my everyday activities
42	Being able to have a social life, like going out to supper with friends, having a drink, going dancing, etc.
43	Experiencing physical pain
44	Not feeling limited by having diabetes
45	Counting carbohydrates for a more flexible diet
46	Worry about going into hospital
47	Feeling overwhelmed by diabetes
48	Pressure from others about whether I'm doing it well or not
49	Worry about hypoglycemias
50	Having illnesses other than diabetes
51	The possibility of developing complications in the future
52	Worry about having high glucose levels
53	Being able to choose the profession I want without being hampered by my diabetes


Subsequently, 54 items were created that covered all the information that the patients had identified as relevant and contained in the 53 issues. This process gave rise to the initial version of the questionnaire, which used Likert type responses (1 = “strongly disagree” to 5 = “strongly agree”).

#### Stage 2: Qualitative analysis of the items, expert review, and refinement of the questionnaire

Once the initial version of the questionnaire was obtained, a qualitative analysis of the items was carried out to determine whether there were any problems of comprehension or any possible ambiguity in their interpretation. To this end, 10 more patients were selected to answer the questionnaire. While they were doing so, they were asked whether they understood the items, whether the items represented their concerns and needs, and so on.

In turn, this initial 54-item version of the questionnaire was evaluated by a group of endocrinologists, nurse educators and psychologists, who identified the items that were potentially ambiguous and might cause confusion, and also the issues that were overrepresented. Each of the items was evaluated and consensus was reached and as a result 14 were eliminated. Thus, this led to a questionnaire comprised of 40 items.

#### Stage 3: Pilot study

To analyze the structure underlying the questionnaire and to obtain information on its psychometric properties, a pilot study of this 40-item version was carried out with 150 patients with type 1 diabetes attending routine visits at the Endocrinology and Nutrition Service of the Complejo Hospitalario Universitario Insular Materno-Infantil in Gran Canaria. As a result of the analyses (see the “Results” Section) the final questionnaire was reduced to 34 items.

#### Stage 4: Psychometric study of the final questionnaire

In this last stage, the psychometric properties of the 34-item questionnaire (hereafter ViDa1) were explored in a sample of 578 patients with type 1 diabetes. In order to contrast the factor model initially obtained in the PCA, a CFA (*N* = 428) was performed using the Unweighted Least Squares Method.

### Procedure

A multicenter study was carried out at seven hospitals in Spain. The investigation was led by the Complejo Hospitalario Universitario Materno-Infantil of Gran Canaria, which recruited the highest number of patients. The study was approved by the Ethics Committees of the corresponding hospitals.

Patients were enrolled at the Endocrinology and Nutrition Services during routine medical visits between February 2014 and May 2016. Participants were informed of the purpose of the study and were invited to participate, then given an informed consent document to complete and sign and issued with a dossier containing the questionnaires detailed in the “Instruments” section.

The patients completed the dossier in the waiting room, where they were able to ask questions or receive help from the researcher. The dossier took ~30 min to complete.

### Participants

Five hundred and ninety-three people with type 1 diabetes were recruited. Fifteen who did not complete all the items of the questionnaire ViDa1 were excluded, and so the final sample consisted of 578 patients, of whom 150 participated in the pilot study.

The following exclusion criteria were applied: age under 14 years (minors are treated at Pediatric Units, and in addition their responses may not reflect their “real” quality of life because the responsibility for the disease is usually shared with their parents); pregnant women, due to the increased self-care characteristic of pregnancy; and all those who could not complete the dossier due to language problems.

The majority of the sample (70.2%) were from the Hospital Universitario Insular Materno Infantil of Gran Canaria, 10.4% from the Hospital Universitario de Gran Canaria Doctor Negrín, 11.4% from the Germans Trias i Pujol University Hospital (Badalona, Barcelona) 3.8% from the Ramón y Cajal University Hospital (Madrid) 2.2% from the University Hospital La Paz (Madrid) 0.5% from Parc Taulí Hospital (Sabadell, Barcelona) and 1.4% from the D-Médical private clinic (Madrid). The age range was 14–71 years and the mean disease duration was 18 years. Table 2 describes the characteristics of the study participants.

**Table 2:** Participants' characteristics. (*N* = 578).

Sex (% women)	41.7
Age (years)^*^	35.2 (11.9)
Duration of illness (years)^*^	18.0 (10)
HbA_1*c*_ (%)^*^	7.9 (1.3)
Insulin treatment (%)	
Multiple doses	77.5
Continuous perfusion pump	17.8
Carbohydrate count (%)	64.2
At least one event (% patients)	
Mild hypoglycemias (per week)	87.7
Severe hypoglycemia (since diagnosis)	33.0
Admission due to hyperglycemia (since diagnosis)	26.6
Psychopharmacological treatment (%)	14.7
Associated cardiovascular risk factors (%)	41.2
Chronic complications (%)	29.6
Retinopathy	23.2
Nephropathy	9.9
Neuropathy	11.1
Macroangiopathy	2.9
Limitation due to complications (%)	8.8
Living arrangements (%)	
With family	72.3
With a partner	18.4
Alone	7.5
Other	1.9
Education (%)	
Illiterate	2.1
Primary education	29.8
Secondary education	39.3
University studies	28.9
Employment status (%)	
Student	17.0
In employment	52.1
Unemployed	23.5
Other	7.4

* *The data are expressed as mean (standard deviation)*.

### Instruments

#### Structured self-administered data collection sheet

This data sheet was designed specifically for the study and it covered the following sociodemographic and clinical variables: sex, age [participants were categorized as adolescents (14–20 years), young (21–30 years), adults (31–50 years), and older (51–71 years)], hospital center, level of education (illiterate, primary, secondary, and university studies), employment situation, living arrangements, duration of disease, type of drug treatment, glycemic control (using the most recent concentration in the last 30 days of glycosylated hemoglobin (HbA_1c_) standardized against NGSP/DCCT), treatment with psychoactive drugs, cardiovascular risk factors (diagnosis of hypertension, dyslipidemia, smoking, and obesity), carbohydrate count, presence, and type of chronic complications and the limitation they represented on participants' daily lives, number of hypoglycemic episodes per week, and presence of acute complications (admissions for severe hyperglycemia or hypoglycemia). Medical variables were confirmed with the patients' medical history.

In addition to the ViDa1 questionnaire, the following instruments were administered:

*Diabetes Quality of Life* (Jacobson et al., 1988) in its adapted and validated version in Spanish (Millán et al., 2002). The instrument is composed of 43 items distributed in four dimensions: satisfaction (15 items), impact (17 items), social/vocational worry (7 items), and diabetes-related worry (4 items). A Likert scale format is used for the responses (1 = “very satisfied” to 5 = “not satisfied” for the satisfaction subscale, and 1 = “never” to 5 = “always” for the others). A total score and scores for each of the different subscales are obtained. Lower scores on the full scale indicate better HRQoL. In our sample Cronbach's alphas were 0.92 for the full questionnaire,0.86 for satisfaction,0.88 for impact,0.78 for social/vocational worry and 0.73 for diabetes-related worry.

*The Satisfaction With Life Scale* (SWLS; Diener et al., 1985). The validated Spanish version was used (Vázquez et al., 2013). Responses are recorded on a Likert scale (0 = “strongly disagree” to 6 = “strongly agree”) and the score range is 0–30. Higher scores indicate higher life satisfaction. The Cronbach's alpha for the total scale in our sample was 0.84.

*The Positive and Negative Affect Schedule* (PANAS; Watson et al., 1988). The validated short form of the schedule was used (Thompson, 2007). This is a self-report measure of 10 items that evaluate negative and positive affect (5 items each). Responses are recorded on a Likert scale (0 = “not at all” to 6 = “totally”) with a score range of 0–30 for each subscale. Higher scores on each subscale indicate a greater increase in positive or negative emotions. Cronbach's alphas in our sample were 0.78 for negative affect and 0.85 for positive affect.

*The General Self-Efficacy Scale* (GSE; Jerusalem and Schwarzer, 1992). The validated Spanish version was administered (Baessler and Schwarcer, 1996). This scale measures respondents' expectations about their ability to cope adequately with a problematic situation. Responses are recorded on a Likert scale (1 = “not at all” to 5 = “totally”) and the score range is 1–50. High scores indicate a higher perception of self-efficacy. In our study Cronbach's alpha for the GSE was 0.89.

*The Hospital Anxiety and Depression Scal*e (HADS; Zigmond and Snaith, 1983). The validated Spanish version was applied (Tejero et al., 1986). This is a self-report measure of 14 items that evaluate symptoms of anxiety and depression (seven items each). Responses are recorded on a Likert scale (0–3) and the score range is 0–21 for each subscale. In our study Cronbach's alpha for anxiety and depression were 0.55 and 0.79, respectively.

*Problem Areas in Diabetes Scale* (PAID; Welch et al., 1997). The validated Spanish-language version was used (Welch et al., 2007). The scale has 20 items which are answered on a Likert scale (0 = “not a problem” at 4 = “a very serious problem”). The scores obtained range from 0 to 100. Higher scores indicate greater emotional distress related to diabetes. In our study the Cronbach's alpha was 0.94.

### Statistical analysis

Data were analyzed using the statistical software SPSS version 23.0, Armonk, New York, IBM Corp. The sampling adequacy was calculated using the Kaiser-Meyer-Olkin (KMO) index and the Bartlett sphericity test, which showed that factor analysis was appropriate for this dataset.

To examine the underlying structure or dimensions of the ViDa1 questionnaire, a Principal Component Analysis (PCA) with orthogonal varimax rotation was performed, since the initial theoretical hypothesis suggested that the factors were independent of each other and that there was no common factor. The relevant information mentioned by the patients was grouped into four different dimensions.

In order to extract the correct number of factors, Kaiser's criterion was used, based on the retention of factors with eigenvalues above 1 (Kaiser, 1958), along with the scree test based on the graphical representation of all eigenvalues (Cattell, 1966) and the initial theoretical hypothesis (Fabrigar et al., 1999).

Items in the pilot study that did not load on any factor, had factor loads lower than 0.40, were overrepresented, or whose elimination improved the Cronbach alpha were eliminated.

Descriptive statistics were calculated for all items (mean, standard deviation, item-total correlation, and item-total correlation if the item was deleted). Reliability was evaluated by calculating the Cronbach's alpha coefficient.

To confirm the four-factor model a Confirmatory Factor Analysis (CFA) was performed in a second sample. CFA is the method most commonly used to obtain evidence of construct validity since it reports the internal structure of the instrument (Zumbo, 2007). CFA was performed with the LISREL version 8.54 program through the Unweighted Least Squares Method; this estimation method was used because it has no limitations with respect to sample size and does not require multivariate normality. It is based on the polychoric correlation matrix and is more suitable for Likert type scales that apply an ordinal scale of measurement (Morata-Ramirez et al., 2015).

The following fit indices were considered: Chi-square/degrees of freedom ratio (χ^2^/_df_: a value <3 was taken to indicate a good fit), the root mean square error of approximation (RMSEA; values close to 0.06 indicate a good fit), the normative fit index (NFI), the non-normalized fit index (NNFI), the comparative fit index (CFI), the goodness-of-fit index (GFI), and the adjusted goodness-of-fit index (AGFI). Values >0.90 suggest a satisfactory fit, and values of 0.95 or higher an optimal fit (Hu and Bentler, 1999).

The study of convergent and discriminant validity was carried out with the Pearson's correlation coefficient following Campbell and Fiske (1959). Those authors argued that, for measures to be valid, the measures of the same construct must correlate highly with each other (convergent validity), and that this correlation must be greater than that between the measures proposed for another construct (discriminant validity). The correlation between HRQoL measured by the various subscales of the ViDa1 questionnaire and the following constructs was studied: HRQoL in diabetes (EsDQOL subscales), satisfaction with life (SWL), self-efficacy (GSE), negative and positive affect (PANAS), anxiety and depression (HADS), and distress due to diabetes (PAID).

ANOVAs and Pearson correlations were performed to provide discriminant validity for the ViDa1 questionnaire in order to determine whether the ViDa1 scores could discriminate between groups that are known to differ in terms of the variable of interest. For the multiple comparisons following statistically significant ANOVAs, the Bonferroni correction was applied to control for the probability of a type I error.

The test-retest reliability (temporal stability) was measured with the Pearson correlation in 95 participants. The sensitivity to change was evaluated with Student's *t*-test for paired samples in 46 subjects. Descriptive statistics were calculated for all quantitative variables (mean and standard deviation) and percentages for categorical variables. All the studied variables presented a normal distribution, as shown by the Kolmogorov-Smirnov test.

## Results

### Pilot study of the ViDa1 questionnaire in 150 patients

The adequacy of the data for PCA was supported by the KMO index, with a value of 0.83, and by the Bartlett sphericity test (χ^2^ = 2771.162; *df* = 780; *p* < 0.001).

The PCA with varimax rotation showed 11 components with eigenvalues above 1 (Kaiser, 1958). However, the scree test (Cattell, 1966) showed an inflection point in the fourth factor. This solution was also supported by the initial theoretical hypothesis (Fabrigar et al., 1999).

Descriptive analyses were performed for each of the 40 items on the ViDa1. Six items that were overrepresented were removed, some because they had negative loadings on all factors and others because their elimination improved the Cronbach's alpha. A new PCA with varimax rotation was performed without the eliminated items and forcing the extraction to four factors. As a result, a 4-factor matrix was obtained with 34 items which accounted for 45% of the total variance. All factors were well defined, since all the factor-relevant items had loadings above 0.40.

### Psychometric study of the ViDa1 questionnaire in 578 patients

#### Dimensions of the questionnaire

The adequacy of the data for PCA was supported by the KMO index (value of 0.89) and by the Bartlett sphericity test (χ^2^ = 5222.845; *df* = 595; *p* < 0.001).

The PCA with varimax rotation showed seven components with eigenvalues above 1 (Kaiser, 1958). However, the scree test (Cattell, 1966) showed an inflection point in the fourth factor. This solution was also supported by the initial theoretical hypothesis and by the results obtained in the pilot study. As a result, it was decided to force the retention to four factors.

As Table 3 shows, the result was a matrix of 34 items with loadings >0.40 on four factors, which account for 45% of the total variance. Although two items had loads of above 0.40 on more than one factor, they were included in the factor in which the loading and theoretical significance were greater.

**Table 3:** Principal Component Analysis with varimax rotation (*N* = 578).

	**Items**			**Components**			
				**1**	**2**	**3**	**4**
1	Having diabetes is a problem for my social relationships (i.e., with friends, work colleagues, partner, etc.)			**0.70**	−0.03	0.18	0.05
2	I feel different because of my diabetes.			**0.68**	0.12	0.10	0.15
3	Having to inject insulin is a daily problem for me.			**0.66**	0.27	−0.13	0.14
4	Having diabetes limits my social life and free time activities (eating out, celebrations, trips, etc.).			**0.65**	0.16	−0.07	0.26
5	My life has been changed by having diabetes.			**0.59**	0.11	0.03	0.26
6	Having diabetes makes my relationship with my family more difficult.			**0.59**	0.03	0.24	0.00
7	I feel limited professionally by my diabetes.			**0.58**	0.00	0.12	0.24
8	In spite of my diabetes I can lead a normal life.			**0.56**	0.36	0.20	0.07
9	One or more complications of my diabetes worsen my quality of life because it limits/they limit me physically.			**0.51**	0.01	0.41	0.16
10	Everyday life with diabetes represents an added source of stress.			**0.49**	0.15	0.26	0.38
11	I worry that other people know that I have diabetes.			**0.48**	0.14	0.04	0.04
12	My sex life is limited by my diabetes.			**0.41**	0.01	0.23	−0.05
13	I am happy with my involvement in the everyday self-care of my diabetes.			0.00	**0.71**	0.34	0.08
14	The level of training/knowledge I have about my diabetes helps me to maintain good control over it.			0.04	**0.69**	0.05	0.05
15	The training I have in carbohydrate counting allows flexibility in my diet.			0.11	**.65**	−0.09	0.08
16	I am happy with the way I cope with my diabetes.			0.06	**0.63**	0.46	0.10
17	I am motivated to take part in the care of my diabetes.			0.04	**0.60**	0.28	−0.01
18	I adjust the insulin dose to my diet to obtain good control.			0.04	**0.60**	0.00	−0.08
19	I am satisfied with my pharmacological treatment because it helps me to control my diabetes.			0.12	0**.59**	0.07	−0.10
20	I am satisfied with my glycemic control at the moment (glycosylated hemoglobin).			−0.10	0**.57**	0.34	0.10
21	The management of my diabetes is a part of my normal everyday life.			0.30	0**.52**	0.25	0.04
22	I consider that I have flexibility and freedom in my diet in spite of my diabetes.			0.25	0**.44**	0.00	0.13
23	I find it hard to carry out the daily controls (glycemias).			0.20	0**.40**	0.12	−0.00
24	I get plenty of rest and I sleep well at night.			0.14	0.14	0**.66**	0.15
25	I feel fine physically.			0.19	0.28	**0.66**	0.04
26	I feel fine psychologically.			0.30	0.26	**0.55**	0.18
27	I have other illnesses as a result of my diabetes which have a negative effect on my quality of life.			0.32	−0.08	**0.50**	0.00
28	I am satisfied with the time I spend doing physical activity.			−0.05	0.27	**0.47**	0.03
29	I think that in general my quality of life is good.			0.31	0.36	**0.49**	0.14
30	I am frightened of having hypoglycemias (fall in sugar level).			0.16	−0.04	0.07	**0.68**
31	I often worry about having a hypoglycemia.			0.20	−0.07	0.05	**0.68**
32	I feel worried when I have high glycemia.			0.04	−0.03	0.00	**0.63**
33	I often worry about having complications in the future due to my diabetes.			0.12	0.09	0.10	**0.67**
34	I often worry about being admitted to hospital because I can't control my diabetes.			0.19	0.26	0.16	**0.53**
	**Factors**	**Eigen value**	**% Variance**	**Accumulated % of Variance**			
	1	8.4	24.9	24.9			
	2	3.4	10.0	35.0			
	3	1.7	5.2	44.2			
	4	1.6	4.7	44.9			

*In bold, the factor weights ≥ 0.40*.

The ViDa1 questionnaire has a multidimensional structure with four main factors: Interference of diabetes in everyday life (composed by 12 items), Self-care (11 items) Well-being (six items), and Worry about the disease (five items).

#### Reliability

Internal consistency was evaluated with Cronbach's alpha, which was 0.86 for the Interference subscale, 0.84 for Self-care, 0.76 for Well-being and 0.71 for Worry. All subscales had coefficients >0.70 and were thus considered adequate (Gliem and Gliem, 2003).

#### Descriptive analysis

Table 4 shows the corrected item-total subscale correlations, which ranged from 0.36 to 0.71. All the items are well represented in the different factors or dimensions of the questionnaire.

**Table 4:** Descriptive analysis of the ViDa1 questionnaire. (*N* = 578).

	***M***	***SD***	**Corrected ítem-total correlation**	**Cronbach's alpha if the item is eliminated**
**INTERFERENCE IN LIFE**				
Having diabetes is a problem for my social relationships (i.e., friends, work colleagues, partner, etc.)	1.7	1.1	0.59	0.85
I feel different because of my diabetes.	2.3	1.3	0.65	0.85
Having to inject insulin is a daily problem for me.	2.5	1.3	0.56	0.85
Having diabetes limits my social life and free time activities (eating out, celebrations, trips, etc.).	2.7	1.4	0.62	0.85
My life has been changed by having diabetes.	3.6	1.3	0.58	0.85
Having diabetes makes my relationship with my family more difficult.	1.7	1.1	0.52	0.85
I feel limited professionally by my diabetes.	2.7	1.4	0.56	0.85
One or more complications of my diabetes worsen my quality of life because it limits/they limit me physically.	2.4	1.3	0.54	0.85
Everyday life with diabetes represents an added source of stress.	3.0	1.3	0.54	0.85
I worry that other people know that I have diabetes.	2.0	1.3	0.58	0.85
My sex life is limited by my diabetes.	2.1	1.2	0.43	0.86
In spite of my diabetes I can lead a normal life.	2.0	1.1	0.38	0.86
**SELF-CARE**				
I am happy with my involvement in the everyday self-care of my diabetes.	3.6	1.1	0.71	0.80
The level of training/knowledge I have about my diabetes helps me to maintain a good control.	4.1	0.9	0.57	0.82
The training I have in carbohydrate counting allows flexibility in my diet.	3.8	1.1	0.47	0.82
I am happy with the way I cope with my diabetes.	3.5	1.1	0.68	0.81
I am motivated to take part in the care of my diabetes.	4.0	1.0	0.57	0.82
I adjust the insulin dose to my diet to obtain good control.	4.4	0.9	0.46	0.82
I am satisfied with my pharmacological treatment because it helps me to control my diabetes.	4.1	1.0	0.49	0.82
I am satisfied with my glycemic control at the moment (glycosylated hemoglobin).	3.0	1.3	0.55	0.82
The management of my diabetes is a part of my normal everyday life	4.1	1.0	0.52	0.82
I consider that I have flexibility and freedom in my diet in spite of my diabetes.	3.6	1.2	0.34	0.84
I find it hard to carry out the daily controls (glycemias).	3.3	1.4	0.36	0.84
**WELL-BEING**				
I get plenty of rest and I sleep well at night.	3.7	1.3	0.55	0.70
I feel fine physically.	3.7	1.2	0.67	0.67
I feel fine psychologically.	3.8	1.2	0.58	0.69
I have other illnesses as a result of my diabetes which have a negative effect on my quality of life.	4.0	1.2	0.30	0.77
I am satisfied with the time I spend doing physical activity.	3.0	1.4	0.54	0.71
I think that in general my quality of life is good.	4.0	0.9	0.37	0.75
**WORRY ABOUT THE DISEASE**				
I am frightened of having hypoglycemias (fall in sugar level).	3.4	1.3	0.51	0.64
I often worry about having a hypoglycemia.	3.7	1.2	0.52	0.64
I feel worried when I have high glycemia.	4.3	0.9	0.52	0.64
I often worry about having complications in the future due to my diabetes.	4.1	1.0	0.36	0.70
I often worry about being admitted to hospital because I can't control my diabetes.	3.2	1.4	0.44	0.68

*M, mean; SD, standard deviation. The items are scored on a Likert scale (1 = strongly disagree, 2 = agree, 3 = neither agree or disagree, 4 = agree, 5 = strongly agree)*.

The multidimensionality of the ViDa1 makes it possible to obtain a total score for each subscale (Table 5) by adding together the scores for each item. For correct interpretation, items 12, 23, and 27 are reversed.

**Table 5:** Descriptive analysis of the ViDa1 according to subscale. (*N* = 578).

	***M***	***SD***	**Mín**.	**Max**.	***N* items**	**Scale**
Interference	29.1	10	12	57	12	(12–60)
Self-care	41.6	7.9	15	55	11	(11–55)
Well-being	22.5	5.1	8	30	6	(6–30)
Worry	19.0	4.1	5	25	5	(5–25)

*M, mean; SD, standard deviation; Min, minimum; Max, maximum*.

Table 6 shows the correlations between the subscales (factors) of the ViDa1.

**Table 6:** Correlations between the ViDa1 subscales (*N* = 578).

	**Self-care**	**Well-being**	**Worry**
Interference	−0.39^**^	−0.53^**^	0.46^**^
Self-care		0.53^**^	−0.20^**^
Well-being			−0.31^**^

** *p < 0.001*.

#### Confirmatory factor analysis (CFA)

In order to contrast the four-factor model initially obtained in the PCA, a CFA (*N* = 428, which did not include the 150 in the pilot study) was performed using the Unweighted Least Squares Method. To determine the goodness of fit of the proposed model, the overall fit indices obtained were interpreted. There were no missing data. The four-factor model developed in the pilot study, achieved optimal fit: the χ^2^/_*df*_ index was 2601.93/554, statistically significant (*p* < 0.0001) and below 3, and the NFI (0.94), NNFI (0.96), IFC (0.96), GFI (0.95), and IFI indices (0.96) were all above.90 (Hu and Bentler, 1999). The RMSEA also presented an appropriate value, not higher than 0.06 (CI = 0.056–0.064; Hu and Bentler, 1999). Since the overall fit indices were adequate, we concluded that the model represents the behavior of the data reasonably well. In summary, the responses of the participants supported the method used to develop the instrument.

#### Convergent-discriminant validity

Convergent-discriminant validity was evaluated through Pearson's correlations between the four subscales of ViDa1 and other related measures.

The four subscales of ViDa1 correlated strongly with the four subscales of another questionnaire on HRQoL in diabetes (EsDQoL) and with the satisfaction with life scale (SWLS). The self-efficacy and positive affect variables also correlated with the subscales of the ViDa1, although more weakly. Anxiety and depression and distress due to diabetes were inversely correlated with the ViDa1 subscales. Correlations between measures are presented in Table 7.

**Table 7:** Correlations between the ViDa1 subscales and other scales (*N* = 578).

	**Interference**	**Self-care**	**Well-being**	**Worry**
Satisfaction (EsDQoL)	0.62^**^	−0.64^**^	−0.68^**^	0.33^**^
Impact (EsDQoL)	−0.84^**^	0.72^**^	0.58^**^	−0.56^**^
Worry: Social/vocational (EsDQoL)	0.45^**^	−0.32^**^	−0.40^**^	0.34^**^
Worry: diabetes-related (EsDQoL)	0.46^**^	−0.35^**^	−0.43^**^	0.56^**^
Satisfaction with life (SWL)	−0.52^**^	0.41^**^	0.55^**^	−0.32^**^
Positive affect (PANAS-P)	−0.41^**^	0.31^**^	0.48^**^	−0.20^**^
Self-efficacy (EAG)	−0.30^**^	0.31^**^	0.36^**^	−0.20^**^
Negative affect (PANAS-N)	0.42^**^	−0.32^**^	−0.45^**^	0.36^**^
Anxiety (HADS-A)	0.45^**^	−0.38^**^	−0.53^**^	0.39^**^
Depression (HADS-D)	0.49^**^	−0.41^**^	−0.61^**^	0.32^**^
Distress due to diabetes (PAID)	0.44^**^	−0.38^**^	−0.34^**^	0.45^**^

** *p < 0.001. In bold, the highest correlations of each subscale on the ViDa1 with the subscales of the EsDQoL. The scores on the EsDQoL are not inverted, and for this reason the correlations are negative*.

#### Known-groups validity

To provide more data on the validity of ViDa1, we explored whether there were differences in the ViDa1 questionnaire scores in certain groups which are known to differ from one another and which are relevant in clinical practice.

As can be seen in Table 8, significant differences were found in the scores of the four subscales of the ViDa1 questionnaire with respect to gender, educational level, age, carbohydrate count in the diet, type of treatment, presence of an additional illness, treatment with psychoactive drugs, and the presence of chronic complications.

**Table 8:** Differences between groups using ANOVA. (*N* = 578).

	***N***	**Int**.	**Self**.	**Well**.	**Worry**.
**Total**	578	29.1 (10)	41.6 (7.9)	22.5 (5.1)	19.0 (4.1)
**SEX**					
Male	241	28.5 (9.9)	41.9 (8.0)	23.8 (4.8)	18.3 (4.2)
Female	337	29.6 (10)	41.4 (7.8)	21.6 (5)	19.4 (4.1)
*F-*value		1.7	0.4	27.2^***^	6.5^**^
**AGE (YEARS)**					
14–20	77	26.9 (10.6)	41.5 (7.8)	24.7 (4.5)	17.5 (4.9)
21–30	126	27.8 (8.9)	40.2 (7.7)	23.1 (4.7)	18.5 (3.7)
31–50	322	30.0 (10)	41.8 (8.1)	21.8 (5.2)	19.3 (4)
51–71	53	30.3 (10.6)	43.5 (7.1)	22.3 (4.5)	19.4 (4.4)
*F-*value		3.0^**^	1.8	7.5^***^	3.1^**^
**EDUCATION**					
Illiterate	12	34.4 (11.7)	37.0 (9.3)	21 (6.9)	20 (3)
Primary studies	172	29.7 (10.5)	40.7 (8.1)	22.2 (5.1)	20.1 (4)
Secondary studies	227	28.9 (9.8)	41.9 (7.9)	22.4 (5.2)	18.7 (4.3)
University studies	167	28.4 (9.5)	42.2 (7.7)	23.2 (4.6)	18.0 (3.9)
*F-*value		1.6	1.4	1.7	5.8^**^
**TREATMENT**					
MDI	448	29.5 (9.7)	41 (7.9)	22.6 (5.1)	18.8 (4.2)
Pump	103	26.5 (10)	44.7 (6.6)	22.4 (4.7)	19.5 (3.8)
Oral injections	27	32.9 (11.9)	38.4 (9.3)	21.2 (6.1)	19.8 (3.7)
*F-*value		5.7^**^	8.5^***^	1	1.1
**CARBOHYDRATE COUNT**					
Yes	370	28.8 (10.1)	43.2 (7.2)	22.9 (4.7)	18.7 (4.2)
No	207	29.7 (9.6)	38.1 (8.3)	21.9 (5.5)	19.5 (4.1)
*F-*value		1.1	42.6^***^	4.4^**^	3.1
**ADDITIONAL ILLNESS**					
Yes	225	31.2 (10)	40.9 (8.1)	20.9 (5.4)	19.7 (4.3)
No	353	29.1 (9.7)	41.9 (7.8)	23.6 (4.5)	18.6 (4)
*F-*value		16^***^	1.4	41.7^***^	6.9^*^
**CHRONIC COMPLICATIONS**					
Yes	171	30.3 (9.8)	41 (8)	20.4 (5.3)	19.5 (3.8)
No	407	28.6 (10)	41.9 (7.9)	23.4 (4.7)	18.7 (4.3)
*F-*value		3.3	1.1	47.5^***^	2.9
**PSYCHOACTIVE DRUGS**					
Yes	85	28.4 (9.1)	39.5 (8.8)	17.8 (4.7)	18.6 (3.7)
No	493	33.0 (10.0)	42.0 (7.6)	23.3 (4.6)	20.5 (4.2)
*F*-value		15.2^***^	6.1^*^	102.6^***^	12.2^**^

Int, Interference; Self, Self-care; Well, Well-being; MDI, multiple daily injections of insulin The table shows means, standard deviations in brackets and the F-value with its significance

* *p < 0.05*.

** *p < 0.01*.

*** *p < 0.001. Significant F-values are highlighted in bold values*.

Bonferroni's *post-hoc* multiple comparisons revealed differences with respect to age in Well-being and Worry. The 14–20 year age group had higher Well-being scores than the 31–50 (*t* = 2.8, *p* < 0.001) and 51–71 year age groups (*t* = 2.4, *p* = 0.042) and lower scores on Worry than the 31–50 year group (*t* = 1.8, *p* = 0.029). Lower scores on Worry were also found in participants who had completed secondary school (*t* = 1.4, *p* = 0.022) and university (*t* = 2.0; *p* < 0.001) than in those who had completed only primary education.

As for type of pharmacological treatment, people on multiple insulin dose treatment presented higher Interference scores than those receiving continuous infusion pump treatment (*t* = 2.9; *p* = 0.021) and lower Self-care scores (*t* = 3.7; *p* < 0.001).

Next, the relationships between the scores on the subscales of ViDa1 with HbA1c and disease duration were studied. HbA_1c_ correlated with scores on Worry *(r* = 0.10, *p* = 0.028) and inversely with Self-care (*r* = −0.39, *p* < 0.001) and Well-being (*r* = −15, *p* < 0.001). Patients with HbA_1c_ of 7% or below had higher scores on Self-care [(45.2 ± 7.2 vs. 40.5 ± 7.8); *t* = 5.2; *p* < 0.001] and lower scores on Worry [(18.1 ± 4.3 vs. 19.2 ± 4.1); *t* = 2.3; *p* < 0.021]. Disease duration correlated inversely with the Well-being subscale (*r* = −17, *p* < 0.001).

#### Temporal stability

Ninety-five patients repeated the ViDa1 questionnaire between 15 and 25 days after their initial participation. The correlations obtained between the initial test and re-test were high for all subscales: Interference (*r* = 0.78, *p* < 0.001), Self-care (*r* = 0.78, *p* < 0.001), Well-being (*r* = 0.77, *p* < 0.001), and Worry (*r* = 0.60, *p* < 0.001).

#### Sensitivity to change

To establish whether the ViDa1 questionnaire was sensitive to detecting changes after a new treatment or after an educational program, 46 patients from the study who completed the questionnaire were assessed 7–15 days before starting the new treatment or program, and then between 30 and 45 days later. Fifteen of these patients received an out-patient, 5-day, structured group therapy education program (the ANAIS program) which promotes a flexible diet with insulin intensification. Another 23 patients started subcutaneous insulin infusion pump treatment and eight, treatment with insulin degludec, a long-acting basal insulin. The ViDa1 subscales Interference [(31.1 ± 9.3 vs. 29.5 ± 9.1); *t* = 9.9; *p* < 0.054] and Self-care [(40.8 ± 6.4 vs. 44.5 ± 6.6); *t* = 4.3; *p* < 0.001)] proved to be sensitive to change.

## Discussion

Type 1 diabetes is a chronic disease with complex therapeutic demands. This high burden of treatment may have a strong impact on patients' lives and their HRQoL.

Two specific instruments are currently available for measuring HRQoL in people with type 1 diabetes, the DQoL and the ADDQoL. However, neither of these questionnaires includes important aspects of patient care such as carbohydrate count in the diet, self-measurement of glucose, and worry about hypoglycemia; they were both developed some 20 or 30 years ago and the treatment of diabetes has evolved since then. In addition, they are designed to measure HRQoL in both type 2 and type 1 diabetes, despite the fact that the two diseases require different therapeutic approaches.

The objective of this study was to develop a new questionnaire for measuring HRQoL in people with type 1 diabetes and to provide information on its psychometric properties. The instrument, the ViDa1, was designed to reflect patients' perceptions of what it means to live with type 1 diabetes, so as to be able to assess the impact of the disease on their lives.

With respect to the questionnaire's psychometric properties, PCA revealed a multidimensional structure with four main dimensions: Interference of diabetes in everyday life, Self-care, Well-being, and Worry about the disease. The questionnaire presented adequate reliability, as evaluated with Cronbach's alpha. All subscales had alpha-values >0.70, as required by current recommendations (Gliem and Gliem, 2003).

The CFA also revealed a good fit of the initially proposed model with the PCA, as indicated by the fit indices (Hu and Bentler, 1999).

As for the questionnaire's convergent-discriminant validity, the associations it presented were as expected. Its subscales correlated highly with the four subscales of the EsDQoL, since both instruments measure the same construct, and also with the satisfaction with life scale (SWLS), another construct previously associated with quality of life (Diener et al., 1985). ViDa1 also correlated inversely with constructs that have been linked with HRQoL such as anxiety and depression (Hassan et al., 2006; Schram et al., 2009) and emotional distress in diabetes (Strandberg et al., 2014).

The ViDa1 questionnaire discriminated between variables that are relevant in the treatment of patients with type 1 diabetes: glycemic control, carbohydrate count, type of insulin treatment, and presence of chronic complications.

A higher HbA_1c_ value was associated with lower scores on Well-being and Self-Care and with higher scores on Interference and Worry. An HbA_1c_ of 7% or less was associated with higher scores on Self-Care and lower scores on Worry. It seems fair to assume that people who involve themselves more in self-care tasks will have lower levels of HbA_1c_; as this indicator has been associated with the development of chronic complications, having a lower HbA_1c_ may be associated with a lower level of worry about this possibility. A better HRQoL has previously been associated with better glycemic control (Hoey et al., 2001; Stahl-Pehe et al., 2017).

Patients who performed carbohydrate counts had higher Self-care and Well-being scores (see Table 8). Indeed, a flexible diet with carbohydrate counts has previously been linked to higher HRQoL (DAFNE Study Group, 2002; Speight et al., 2010).

Although there is some controversy regarding the association between HRQoL and insulin pump treatment (Barnard et al., 2007) our results suggest that patients receiving this therapy reported higher scores on Self-care and lower scores on Interference; that is, they experience fewer limitations due to their diabetes. Other studies have also found higher HRQoL in people with this form of insulin administration (Nuboer et al., 2008; Ortiz et al., 2010).

Patients with chronic complications scored lower on the Well-being subscale. Other authors have already reported that chronic complications decrease HRQoL in patients with type 1 diabetes (Hahl et al., 2002; Jacobson et al., 2013).

ViDa1 also contributed information regarding other subgroups of interest. Adolescents presented higher scores on Well-being and lower scores on Worry, in accordance with previous results (Millán et al., 2002). Women had lower scores on Well-being and higher scores on Worry than men. Other authors using the DQOL reported a greater impact and higher degree of worry regarding diabetes among women (Millán et al., 2002).

As for the temporal stability of the ViDa1 questionnaire, the high correlations found in the retest suggest that it has good test-retest reliability. The questionnaire also demonstrated its sensitivity to change on the Self-care (*t* = 4.3; *p* < 0.001) and Interference subscales (*t* = 1.9; *p* = 0.054) after an educational program that promoted a flexible diet or after switching to a new insulin treatment.

Among the main strengths of this study is the participation of the patients themselves in delimiting the concept of HRQoL and in developing the questionnaire, and the assessment of its psychometric properties in a heterogeneous sample of patients from many different regions of Spain. However, we are aware that the study also has limitations. Firstly, HRQoL is a complex construct, which is particularly difficult to measure. In the second place, for PCA, we used orthogonal rotation. We are aware that most phenomena assessed in health studies are interrelated, and that perfect othogonality is difficult to find. The correlations found between the factors obtained were relatively low and the solution obtained with othogonal rotation was simple informative and clinically applicable. In addition, only a small sample of patients (*N* = 46) and three types of intervention were used to measure sensitivity to change. One way of providing more evidence regarding the questionnaire's sensitivity to change would be to carry out a longitudinal follow-up that records several measures over time. Additional information could also be provided by a specific study in adolescents, since only 52 subjects were aged between 14 and 17 years. Finally, the questionnaire is in Spanish; although this is the second most spoken language in the world, a large number of patients will be unable to complete it at present. In the future we hope to be able to make adaptations and validations of the instrument in other languages so as to extend its use.

## Conclusion

The ViDa1 questionnaire is a new instrument for measuring HRQoL in people with type 1 diabetes. It covers the most important aspects of what it means to live with this disease through its four dimensions: Interference of diabetes in life, Self-care, Well-being, and Worry about the disease.

Our results suggest that the ViDa1 questionnaire has adequate psychometric properties and may be a useful tool for evaluating the fluctuations in the disease that patients experience over their lifetime, as well as for measuring the impact of specific education programs.

## Ethics statement

This study was carried out in accordance with the recommendations of Local Ethics Committee with written informed consent from all subjects. All subjects gave written informed consent in accordance with the Declaration of Helsinki. The protocol was approved by the CEIC Hospital Universitario Insular Materno-Infantil de Gran Canaria.

## Author contributions

DA designed the study, interviewed the patients, collected, analyzed and interpreted the data, and wrote the manuscript. MR participated in the design of the study, the creation of the questionnaire, data analysis, interpretation of results, and review of the article. MC interviewed patients and reviewed medical records. AC and MB participated in the development of the questionnaire, patient recruitment, data collection, and manuscript review. AE transferred data. LN, MP, PL, NH, PM, DS, LS and recruited patients and reviewed medical records. AS and DM reviewed medical records. AW participated in the design of the study, development of the questionnaire, patient recruitment, data collection, interpretation of the results, and manuscript review. PdP-V and FN participated in data collection and critical review of the manuscript.

### Conflict of interest statement

The authors declare that the research was conducted in the absence of any commercial or financial relationships that could be construed as a potential conflict of interest.

## References

[B1] Alcubierre N. Rubinat E. Traveset A. Martinez-Alonso M. Hernandez M. Jurjo C.. (2014). A prospective cross-sectional study on quality of life and treatment satisfaction in type 2 diabetic patients with retinopathy without other major late diabetic complications. Health Qual. Life Outcomes 12:131. 10.1186/s12955-014-0131-2 25138117 PMC4244048

[B2] Alvarado-Martel D. Cañas F. Velasco R. Alcubierre N. López-Ríos L. Rius F.. (2015a). Design, construction, and implementation of an online platform for patients with type 1 diabetes: EncoDiab. Patient Prefer. Adherence 9, 767–775. 10.2147/PPA.S77730 26124644 PMC4476477

[B3] Alvarado-Martel D. Velasco R. Sánchez-Hernández R. M. Carrillo A. Nóvoa F. J. Wägner A. M. (2015b). Quality of life and type 1 diabetes: a study assessing patients' perceptions and self-management needs. Patient Prefer. Adherence 9, 1315–1323. 10.2147/PPA.S87310 26396503 PMC4576890

[B4] Atkinson M. A. Eisenbarth G. S. Michels A. W. (2014). Type 1 diabetes. Lancet 383, 69–82. 10.1016/S0140-6736(13)60591-7 23890997 PMC4380133

[B5] Baessler R. Schwarcer J. (1996). Evaluación de la autoeficacia: adaptación española de la escala de Autoeficacia General. Ansiedad y Estrés 2, 1–8.

[B6] Barnard K. D. Lloyd C. E. Skinner T. C. (2007). Systematic literature review: quality of life associated with insulin pump use in type 1 diabetes. Diabet. Med. 24, 607–617. 10.1111/j.1464-5491.2007.02120.x 17367304

[B7] Botija Yagüe M. P. Lizán Tudela L. Gosalbes Soler V. Bonet Plá Á. Fornos Garrigós A. (2007). How does intensive therapy to control cardiovascular risk factors affect health-related quality of life in diabetic patients? | ¿Cómo influye el tratamiento intensivo de los factores de riesgo cardiovascular en la calidad de vida relacionada con la salud. Aten. Primaria 39, 227–233. 10.1157/13101795 17493446 PMC7659570

[B8] Bradley C. Todd C. Gorton T. Symonds E. Martin A. Plowright R. (1999). The development of an individualized questionnaire measure of perceived impact of diabetes on quality of life: the ADDQoL. Qual. Life Res. 8, 79–91. 10.1023/A:1026485130100 10457741

[B9] Byrne M. Newell J. Coffey N. O'Hara M. C. Cooke D. Dinneen S. F. (2012). Predictors of quality of life gains among people with type 1 diabetes participating in the Dose Adjustment for Normal Eating (DAFNE) structured education programme. Diabetes Res. Clin. Pract. 98, 243–248. 10.1016/j.diabres.2012.09.017 23018180

[B10] Campbell D. T. Fiske D. W. (1959). Convergent and discriminant validation by the multitrait-multimethod matrix. Psychol. Bull. 56, 81–105. 10.1037/h0046016 13634291

[B11] Cattell R. B. (1966). The scree test for the number of factors. Multivariate Behav. Res. 1, 245–276. 10.1207/s15327906mbr0102_10 26828106

[B12] Cooke D. Bond R. Lawton J. Rankin D. Heller S. Clark M.. (2015). Modeling predictors of changes in glycemic control and diabetes-specific quality of life amongst adults with type 1 diabetes 1 year after structured education in flexible, intensive insulin therapy. J. Behav. Med. 38, 817–829. 10.1007/s10865-015-9649-y 26072044

[B13] Correr C. J. Pontarolo R. Melchiors A. C. Rossignoli P. Fernández-Llimós F. Radominski R. B. (2008). Translation to Portuguese and validation of the diabetes quality of life measure (DQOL-Brazil) | Tradução para o Português e validação do instrumento diabetes quality of life measure (DQOL-Brasil). Arq. Bras. Endocrinol. Metabol. 52, 515–522. 10.1590/S0004-27302008000300012 18506277

[B14] da Costa F. A. Guerreiro J. P. Duggan C. (2006). An Audit of Diabetes Dependent Quality of Life (ADDQoL) for Portugal: exploring validity and reliability | Análisis de la calidad de vida relacionada con la diabetes (ADDQoL) para Portugal: Exploración de la validez y la fiabilidad. Pharm. Pract. 4, 123–128. 10.4321/S.1885-642X.2006000300004 PMC4156844 25214898

[B15] DAFNE Study Group (2002). Training in flexible, intensive insulin management to enable dietary freedom in people with type 1 diabetes: dose adjustment for normal eating (DAFNE) randomised controlled trial. BMJ 325:746. 10.1136/bmj.325.7367.746 12364302 PMC128375

[B16] DePablos-Velasco P. Salguero-Chaves E. Q. Mata-Poyo J. DeRivas-Otero B. García-Sánchez R. Viguera-Ester P. (2014). Quality of life and satisfaction with treatment in subjects with type 2 diabetes: results in Spain of the PANORAMA study | Calidad de vida y satisfacción con el tratamiento de sujetos con diabetes tipo 2: resultados en Espa-a del estudio PANORAMA. Endocrinol. y Nutr. 61, 18–26. 10.1016/j.endonu.2013.05.005 24055176

[B17] Diabetes Control Complications Trial Research Group (1993). The effect of intensive treatment of diabetes on the development and progression of long-term complications in insulin-dependent diabetes mellitus. Diabetes Control and Complications Trial Research Group. N. Engl. J. Med. 329, 977–986. 10.1056/NEJM199309303291401 8366922

[B18] Diabetes Control Complications Trial Research Group (1996). Influence of intensive diabetes treatment on quality-of-life outcomes in the diabetes control and complications trial. Diabetes Care 19, 195–203. 10.2337/diacare.19.3.195 8742561

[B19] Diener E. Emmons R. A. Larsen R. J. Griffin S. (1985). The satisfaction with life scale. J. Pers. Assess. 49, 71–75. 10.1207/s15327752jpa4901_13 16367493

[B20] Escudero-Carretero M. J. Prieto-Rodríguez M. Á. Fernández-Fernández I. March-Cerdá J. C. (2007). Expectations held by type 1 and 2 diabetes mellitus patients and their relatives: the importance of facilitating the health-care process. Health Expect. 10, 337–349. 10.1111/j.1369-7625.2007.00452.x 17986070 PMC5060416

[B21] Fabrigar L. R. MacCallum R. C. Wegener D. T. Strahan E. J. (1999). Evaluating the use of exploratory factor analysis in psychological research. Psychol. Methods 4, 272–299. 10.1037/1082-989X.4.3.272

[B22] Fung C. S. C. Wan E. Y. F. Yu C. L. Y. Wong C. K. H. (2016). Validity and reliability of the 19-item Audit of Diabetes-Dependent Quality of Life (ADDQoL-19) questionnaire in Chinese patients with type 2 diabetes mellitus in primary care. Qual. Life Res. 25, 2373–2378. 10.1007/s11136-016-1263-0 26980420

[B23] Gibbons E. Fitzpatrick R. (2009). A Structured Review of Patient-Reported Outcome Measures (PROMs) for Diabetes. Oxford: Patient Reported Outcome Measurement Group, University of Oxford.

[B24] Gliem J. A. Gliem R. R. (2003). Calculating, interpreting, and reporting Cronbach's alpha reliability coefficient for Likert-type scales. Pap. Present. Midwest Res. Pract. Conf. Adult, Contin. Community Educ. Columbus. 82–88.

[B25] Hahl J. Hämäläinen H. Sintonen H. Simell T. Arinen S. Simell O. (2002). Health-related quality of life in type 1 diabetes without or with symptoms of long-term complications. Qual. Life Res. 11, 427–436. 10.1023/A:1015684100227 12113390

[B26] Hassan K. Loar R. Anderson B. J. Heptulla R. A. (2006). The role of socioeconomic status, depression, quality of life, and glycemic control in type 1 diabetes mellitus. J. Pediatr. 149, 526–531. 10.1016/j.jpeds.2006.05.039 17011326

[B27] Hirose A. S. Fujihara K. Miyamasu F. Iwakabe S. Shimpo M. Heianza Y. (2016). Development and evaluation of the Japanese version of the audit of diabetes-dependent quality of life for patients with diabetes. Diabetol. Int. 7, 384–390. 10.1007/s13340-016-0260-4 PMC6224971 30603290

[B28] Hoey H. Aanstoot H.-J. Chiarelli F. Daneman D. Danne T. Dorchy H.. (2001). Good metabolic control is associated with better quality of life in 2,101 adolescents with type 1 diabetes. Diabetes Care 24, 1923–1928. 10.2337/diacare.24.11.1923 11679458

[B29] Hu L.-T. Bentler P. M. (1999). Cutoff criteria for fit indexes in covariance structure analysis: conventional criteria versus new alternatives. Struct. Equ. Model. 6, 1–55. 10.1080/10705519909540118

[B30] Huang I.-C. Hwang C.-C. Wu M.-Y. Lin W. Leite W. Wu A. W. (2008). Diabetes-specific or generic measures for health-related quality of life? Evidence from psychometric validation of the D-39 and SF-36. Value Health 11, 450–461. 10.1111/j.1524-4733.2007.00261.x 18489668

[B31] Jacobson A. Barofsky I. Cleary P. Rand L. (1988). Reliability and validity of a diabetes quality-of-life measure of the Diabetes Control and Complications Trial (DCCT). Diabetes Care 11, 725–732. 10.2337/diacare.11.9.725 3066604

[B32] Jacobson A. M. Braffett B. H. Cleary P. A. Gubitosi-Klug R. A. Larkin M. E. (2013). The long-term effects of type 1 diabetes treatment and complications on health-related quality of life: a 23-year follow-up of the diabetes control and complications/epidemiology of diabetes interventions and complications cohort. Diabetes Care 36, 3131–3138. 10.2337/dc12-2109 23835693 PMC3781542

[B33] Jerusalem M. Schwarzer R. (1992). Self-efficacy as a resource factor in stress appraisal procecesses, in Self-Efficacy: Thought Control of Action, ed Schwarzer R. (Washington, DC: Hemisphere), 195–213.

[B34] Kaiser H. F. (1958). The varimax criterion for analytic rotation in factor analysis. Psychometrika 23, 187–200. 10.1007/BF02289233

[B35] Kong D. Ding Y. Zuo X. Su W. Xiu L. Lin M.. (2011). Adaptation of the audit of diabetes-dependent quality of life questionnaire to people with diabetes in China. Diabetes Res. Clin. Pract. 94, 45–52. 10.1016/j.diabres.2011.05.026 21683467

[B36] Millán M. M. Reviriego J. Del Campo J. (2002). Revaluación de la versión espa-ola del cuestionario Diabetes Quality of Life (EsDQOL). Endocrinol. y Nutr. 49, 322–324. 10.1016/S1575-0922(02)74482-3

[B37] Morata-Ramirez M. Á. Holgado Tello F. P. Barbero-García M. I. Mendez G. (2015). Análisis factorial confirmatorio. Recomendaciones sobre mínimos cuadrados no ponderados en función del error Tipo I de Ji-Cuadrado y RMSEA [Confirmatory factor analysis. Recommendations for unweighted least squares method related to Chi-Square and RMSEA]. Acción Psicol. 12, 7–90. 10.5944/ap.12.1.14362

[B38] Nuboer R. Borsboom G. J. J. M. Zoethout J. A. Koot H. M. Bruining J. (2008). Effects of insulin pump vs. injection treatment on quality of life and impact of disease in children with type 1 diabetes mellitus in a randomized, prospective comparison. Pediatr. Diabetes 9, 291–296. 10.1111/j.1399-5448.2008.00396.x 18466210

[B39] Ortiz M. T. A. Díaz F. F. C. Romero A. M. Ruiz de Adana Navas M. S. Domínguez-López M. Gonzalo-Marín M. (2010). Impact of intensive therapy with continuous subcutaneous insulin infusion on quality of life in patients with type 1 diabetes. J. Appl. Biobehav. Res. 15, 1–19. 10.1111/j.1751-9861.2010.00049.x

[B40] Ostini R. Dower J. Donald M. (2012). The audit of Diabetes-Dependent Quality of Life 19 (ADDQoL): feasibility, reliability and validity in a population-based sample of Australian adults. Qual. Life Res. 21, 1471–1477. 10.1007/s11136-011-0043-0 22012024

[B41] Pakpour A. H. Saffari M. Burri A. (2012). Translation and validation of an Iranian version of the diabetes quality of life measure. J. Diabetes Investig. 3, 471–478. 10.1111/j.2040-1124.2012.00217.x 24843609 PMC4019249

[B42] Perrotta C. Irazola V. (2002). Validación linguistica de un cuestionario específico para medir calidad de vida relacionada con la salud (CVRS) ‘Audit of life diabetes dependent questionnaire’ (ADDQOL) al español como se habla en la Argentina. Rev. Soc. Arg. Diabetes 36:107.

[B43] Schram M. T. Baan C. A. Pouwer F. (2009). Diabetes patients are known to have a worse quality of life than individuals without diabetes. They also have an increased risk for depressive symptoms, which may have an additional negative effect on their quality of life. This systematic review summariz. Curr. Diabetes Rev. 5, 112–119. 10.2174/157339909788166828 19442096 PMC2764861

[B44] Sparring V. Nyström L. Wahlström R. Jonsson P. M. Ostman J. Burström K. (2013). Diabetes duration and health-related quality of life in individuals with onset of diabetes in the age group 15-34 years - a Swedish population-based study using EQ-5D. BMC Public Health 13:377. 10.1186/1471-2458-13-377 23607813 PMC3640903

[B45] Speight J. Amiel S. A. Bradley C. Heller S. Oliver L. Roberts S.. (2010). Long-term biomedical and psychosocial outcomes following DAFNE (Dose Adjustment For Normal Eating) structured education to promote intensive insulin therapy in adults with sub-optimally controlled Type 1 diabetes. Diabetes Res. Clin. Pract. 89, 22–29. 10.1016/j.diabres.2010.03.017 20399523

[B46] Speight J. Reaney M. D. Barnard K. D. (2009). Not all roads lead to Rome-a review of quality of life measurement in adults with diabetes. Diabet. Med. 26, 315–327. 10.1111/j.1464-5491.2009.02682.x 19388959

[B47] Stahl-Pehe A. Landwehr S. Lange K. S. Bächle C. Castillo K. Yossa R.. (2017). Impact of quality of life (QoL) on glycemic control (HbA1c) among adolescents and emerging adults with long-duration type 1 diabetes: a prospective cohort-study. Pediatr. Diabetes [Epub ahead of print]. 10.1111/pedi.12487 28133885

[B48] Strandberg R. B. Graue M. Wentzel-Larsen T. Peyrot M. Rokne B. (2014). Relationships of diabetes-specific emotional distress, depression, anxiety, and overall well-being with HbA1c in adult persons with type 1 diabetes. J. Psychosom. Res. 77, 174–179. 10.1016/j.jpsychores.2014.06.015 25149027

[B49] Sundaram M. Kavookjian J. Patrick J. H. Miller L.-A. Suresh Madhavan S. Scott V. (2007). Quality of life, health status and clinical outcomes in Type 2 diabetes patients. Qual. Life Res. 16, 165–177. 10.1007/s11136-006-9105-0 17033903

[B50] Tan S. M. K. Shafiee Z. Wu L. L. Rizal A. M. Rey J. M. (2005). Factors associated with control of type I diabetes in Malaysian adolescents and young adults. Int. J. Psychiatry Med. 35, 123–136. 10.2190/EQ71-RMWV-6CEJ-1DGM 16240970

[B51] Testa M. A. Simonson D. C. (1996). Assesment of quality-of-life outcomes. N. Engl. J. Med. 334, 835–840. 10.1056/NEJM199603283341306 8596551

[B52] Tejero A. Guimerá E. M. Farré J. M. Peri J. (1986). Uso clínico del HAD (Hospital Anxiety and Depression Scale) en poblaciÃşn psiquiátrica: un estudio de su sensibilidad, fiabilidad y validez. Rev. Depto Psiquiatría Facultad de Med Barna 13, 233–238.

[B53] The Food Drug Administration (2006). Guidance for industry: patient-reported outcome measures: use in medical product development to support labeling claims: draft guidance. Health Qual. Life Outcomes 4:79. 10.1186/1477-7525-4-79 17034633 PMC1629006

[B54] Thompson E. R. (2007). Development and validation of an internationally reliable short-form of the Positive and Negative Affect Schedule (PANAS). J. Cross. Cult. Psychol. 38, 227–242. 10.1177/0022022106297301

[B55] Tsui E. Barnie A. Ross S. Parkes R. Zinman B. (2001). Intensive insulin therapy with insulin lispro: a randomized trial of continuous subcutaneous insulin infusion versus multiple daily insulin injection. Diabetes Care 24, 1722–1727. 10.2337/diacare.24.10.1722 11574432

[B56] Vázquez C. Duque A. Hervás G. (2013). Satisfaction with life scale in a representative sample of Spanish adults: validation and normative data. Span. J. Psychol. 16:E82. 10.1017/sjp.2013.82 24230945

[B57] Watson D. Clark L. A. Tellegen A. (1988). Development and validation of brief measures of positive and negative affect: the PANAS scales. J. Pers. Soc. Psychol. 54, 1063–1070. 10.1037/0022-3514.54.6.1063 3397865

[B58] Welch G. Schwartz C. E. Santiago-Kelly P. Garb J. Shayne R. Bode R. (2007). Disease-related emotional distress of Hispanic and non-Hispanic type 2 diabetes patients. *Ethn*. Dis. 17, 541–547. 17985511

[B59] Welch G. W. Jacobson A. M. Polonsky W. H. (1997). The problem areas in diabetes scale. An evaluation of its clinical utility. Diabetes Care 20, 760–766. 10.2337/diacare.20.5.760 9135939

[B60] Wolpert H. A. Anderson B. J. (2001). Management of diabetes: are doctors framing the benefits from the wrong perspective? Br. Med. J. 323, 994–996. 10.1136/bmj.323.7319.994 11679393 PMC1121514

[B61] Zigmond A. S. Snaith R. P. (1983). The hospital anxiety and depression scale. Acta Psychiatr. Scand. 67, 361–370. 10.1111/j.1600-0447.1983.tb09716.x 6880820

[B62] Zumbo B. D. (2007). Validity: foundational issues and statistical methodology, in Handbook of Statistics. Psychometrics, eds Rao C. R. Sinharay S. (Amsterdam: Elsevier), 45–79.

